# Properties of Probiotic Bacterial Cellulose/κ-Carrageenan Based Hydrogel Having Antibacterial Activity and Biocompatibility

**DOI:** 10.3390/gels12050353

**Published:** 2026-04-23

**Authors:** Mainak Chaudhuri, Nabanita Saha, Arita Dubnika, Petr Sáha

**Affiliations:** 1 Centre of Polymer Systems, Tomas Bata University in Zlin, 760 01 Zlin, Czech Republic; chaudhuri@utb.cz (M.C.); saha@utb.cz (P.S.); 2 Institute of Biomaterials and Bioengineering, Faculty of Natural Sciences and Technology, Riga Technical University, LV-1048 Riga, Latvia; arita.dubnika@rtu.lv; 3 Baltic Biomaterials Centre of Excellence, Headquarters at Riga Technical University, LV-1048 Riga, Latvia

**Keywords:** probiotic bacterial cellulose, κ-carrageenan, biopolymer, hydrogel, antibacterial, biocompatible, biomedical

## Abstract

Hydrogels derived from biopolymers have attracted considerable interest in biomedical applications because of their biocompatibility and structural similarity to the extracellular matrix (ECM). Bacterial Cellulose (BC), despite being a promising biopolymer for hydrogel preparation, lacks antimicrobial properties itself. To address this drawback, we prepared Probiotic Bacterial Cellulose (PBC) in our laboratory, which has intrinsic antibacterial properties. No research was found on the preparation of a hydrogel using PBC and κ-carrageenan, which motivated us to develop a PBC/κ-carrageenan-based hydrogel. In the study, a novel biocomposite hydrogel system has been developed by integrating PBC with κ-carrageenan, yielding a multifunctional hydrogel with enhanced antibacterial properties and biocompatibility. The novel hydrogel has been evaluated for its structural, physicochemical, antibacterial, and biocompatible properties. Fourier transform infrared spectroscopy (FTIR) analysis confirmed the formation of intermolecular interactions between PBC and κ-carrageenan. Scanning electron microscopy (SEM) images revealed a porous internal morphology and the presence of probiotic bacteria within the hydrogel networks. Porosity analysis and swelling behaviour indicated an elevated water uptake capacity and structural stability. The composite hydrogel demonstrated promising antibacterial properties against pathogenic bacteria *Escherichia coli* (Gram-negative) and *Staphylococcus aureus* (Gram-positive) and exhibited favourable in vitro biocompatibility. The developed PBC/κ-carrageenan hydrogel exhibits a synergistic combination of porosity, swelling capacity, biocompatibility, and antibacterial activity, making it a potential candidate for healthcare applications viz. wound healing and other tissue engineering applications.

## 1. Introduction

Cellulose-based hydrogels have become a well-known class of biomaterials due to their superior biocompatibility and biodegradability, and because they are obtained from renewable sources. The 3D network structure of these materials is characterized by a high degree of hydration, which facilitates effective gas exchange, cellular connections, and nutrient diffusion. This structural similarity to the ECM of biological tissues is a key property that contributes to their biological functionality [1]. These qualities are highly suitable for various biomedical applications such as tissue engineering, drug delivery, and wound healing. BC is a naturally occurring biopolymer that has gained considerable attention for its high purity, extraordinary mechanical strength, and distinctive nanofibrous architecture, which provides a substantial surface area and a remarkable water-retention capacity [2].

Notwithstanding the aforementioned benefits, native BC lacks inherent antibacterial activity [3], a limitation that restricts its utilization in environments susceptible to infection, such as wound beds [4]. It is imperative to recognize the significance of effective infection control in wound care, given the potential impediments to tissue regeneration, and the severe consequences of microbial contamination [5]. Consequently, the development of cellulose-based hydrogels with intrinsic antibacterial properties remains a major focus of research in this field.

In order to overcome these limitations, several approaches have been investigated, including the addition of antimicrobial agents to BC-based systems, such as metal nanoparticles [6], antibiotics [7], and bioactive substances [8]. However, these methods often have drawbacks, including the potential for toxicity, reduced long-term stability, and the potential to foster antibiotic resistance. Probiotic-based approaches have recently garnered interest as a more sustainable and safe option in this regard [9]. The co-culturing of cellulose-producing bacteria with probiotic strains has been shown to result in the production of PBC, which has been demonstrated to provide a novel technique to impart inherent antibacterial capabilities [10]. The addition of probiotic microorganisms, such as *Lactobacillus plantarum* and *Pediococcus pentosaceus*, results in the production of antimicrobial metabolites such as bacteriocins and organic acids, which have the capacity to impede the proliferation of deleterious bacteria. These metabolites are produced in situ by bacterial metabolism [11].

Several studies demonstrated probiotic encapsulation inside the hydrogel network as an effective delivery system due to its ability to protect microorganisms and enhance their viability [12]. Probiotic encapsulation has been the subject of extensive research, employing conventional hydrogel systems derived from natural polymers, including alginate, chitosan, pectin, and κ-carrageenan. These systems have demonstrated enhanced survivability and regulated release behaviour under conditions that mimic physiological settings [13]. The protective barrier that these hydrogels establish functions to shield probiotic cells from adverse environmental factors, including bile salts and the highly acidic conditions present within the stomach [14]. However, a significant number of these systems depend on post-encapsulation techniques and frequently exhibit drawbacks such as inadequate mechanical stability, early release, and poor long-term viable cell protection [13]. In this regard, a unique method has been developed that incorporates in situ probiotic integration inside a strong nanofibrous cellulose network. This method is referred to as probiotic bacterial cellulose (PBC), which is produced by co-culturing probiotic strains with *Komagataeibacter xylinus*. This approach, in contrast to traditional encapsulation techniques, facilitates the creation of a highly porous and structurally stable matrix that can both sustain probiotic life and offer controlled release [8]. Also, the beneficial properties of probiotic bacteria and their derivatives get incorporated inside the BC network while synthesizing the PBC [10].

Concurrently, κ-carrageenan, a sulphated polysaccharide derived from red seaweed (*Kappaphycus alvarezii*), has been extensively utilized in hydrogel systems due to its superior gel-forming ability, biocompatibility, and potential for ionic crosslinking in the presence of potassium ions (K^+^) [15]. The integration of κ-carrageenan with cellulose-based materials provides a flexible platform for creating composite hydrogels with adjustable physicochemical characteristics, including improved swelling behaviour, variable porosity, and enhanced structural stability. In the context of biomedical applications, where precise control over material properties is paramount, such composite systems prove to be highly advantageous.

Although research focusing on incorporating probiotic bacterial cellulose (PBC) into κ-carrageenan-based hydrogel systems is limited, previous studies have documented the use of BC/κ-carrageenan composite hydrogels [16]. Specifically, the combined impact of ionic crosslinking and probiotic activity on the structural, physicochemical, antibacterial, and cytocompatibility properties of these hydrogels has not yet been the subject of a thorough study. Previous studies have demonstrated that BC-based hydrogel systems have been developed for potential biomedical applications due to their porous, non-toxic, biocompatible nature [17]. In order to advance the design of multifunctional cellulose-based biomaterials, it is essential to address this gap in current research. The novelty of the present study lies in the preparation of a PBC-based hydrogel system that combines biosynthesis-driven encapsulation of probiotic bacteria and their derivatives with another biopolymer, κ-carrageenan, with enhanced structural integrity, offering a promising alternative to traditional probiotic-loaded hydrogels.

Therefore, this study focuses on developing a novel PBC/κ-carrageenan composite hydrogel that exhibits enhanced biocompatibility and antibacterial activity. In order to comprehensively evaluate the impact of composition and crosslinking on the hydrogels’ characteristics, a range of polymer ratios was used in their fabrication, both with and without potassium ion-mediated ionic crosslinking. The structural characteristics, swelling behaviour, porosity, gel fraction, antiseptic loading capacity, antibacterial activity against *Staphylococcus aureus* and *Escherichia coli*, and in vitro cytocompatibility of the produced hydrogels were all evaluated. The findings of this study demonstrate the potential of PBC-based hydrogels for biomedical applications, such as wound healing and tissue engineering, and offer significant insights into their design.

## 2. Results and Discussion

### 2.1. Preparation of Hydrogel Samples

**Figure 1:**
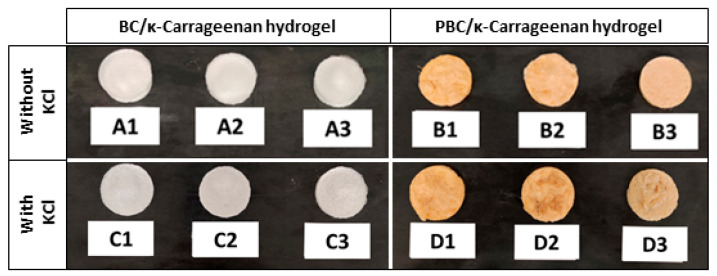
Freeze-dried BC/κ-carrageenan (A1–A3 and B1–B3) and PBC/κ-carrageenan (C1–C3 and D1–D3) hydrogels.

Figure 1 shows the freeze-dried hydrogel samples, where BC/κ-carrageenan hydrogels appear white in colour and PBC/κ-carrageenan hydrogels appear light brown in colour. Also, with a decrease in the PBC ratio, the colour of the hydrogels with higher κ-carrageenan concentration becomes lighter than that of the hydrogels with higher PBC concentration.

### 2.2. FTIR Analysis of PBC/κ-Carrageenan Hydrogels

**Figure 2:**
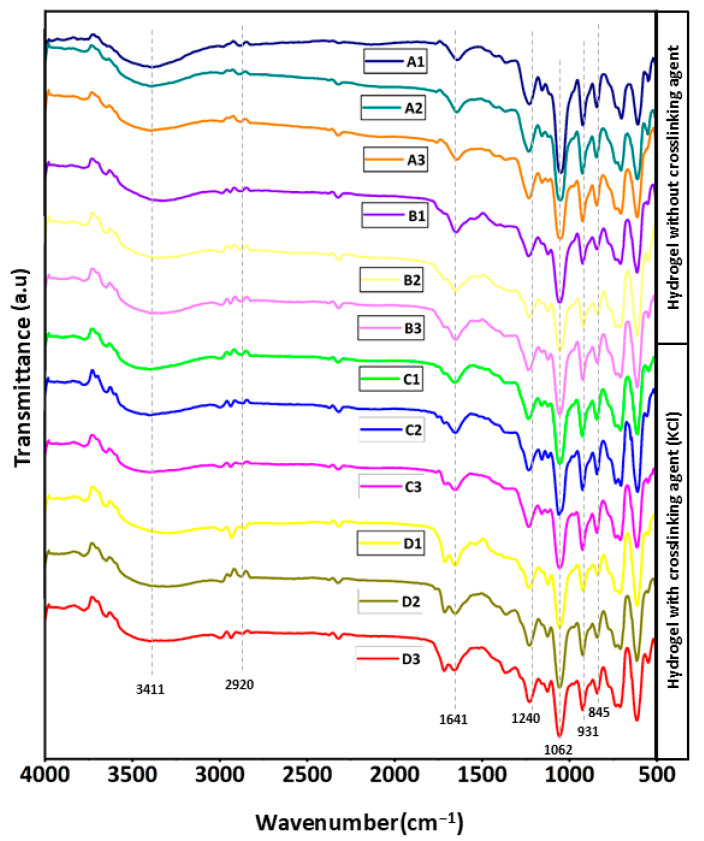
FTIR spectra of BC/κ-carrageenan and PBC/κ-carrageenan hydrogel samples (A1–A3 = BC/κ-carrageenan hydrogel without crosslinker, B1–B3 = PBC/κ-carrageenan hydrogel without crosslinker, C1–C3 = BC/κ-carrageenan hydrogel with crosslinker, D1–D3 = PBC/κ-carrageenan hydrogel with crosslinker).

FTIR spectroscopy was used to investigate the chemical structure and interactions within the prepared hydrogels, as demonstrated in Figure 2. All hydrogel samples exhibited a broad band around 3200–3400 cm^−1^, which corresponds to O-H stretching vibrations of the hydroxyl groups in the cellulose and κ-carrageenan polymers. This indicates the presence of hydrogen bonding within the hydrogel network [18]. Peaks observed near 2920 cm^−1^ were attributed to C-H stretching of the polysaccharide backbone [19]. A band around 1640 cm^−1^ referred to the bending vibration of absorbed water molecules associated with the hydrophilic matrix [20]. The characteristic peak of κ-carrageenan at ~1220 cm^−1^ corresponds to sulphate ester (O=S=O) stretching, confirming the presence of sulphated groups from κ-carrageenan [21]. Additionally, strong bands between 1000 and 1100 cm^−1^ were attributed to C–O–C and C–O stretching vibrations of glycosidic bonds [22]. Peaks at ~930 and ~845 cm^−1^ correspond to 3,6-anhydro-D-galactose and D-galactose-4-sulphate, respectively, which are characteristic of κ-carrageenan [23].

All hydrogel samples showed similar spectral characteristics, suggesting that adding BC/PBC with κ-carrageenan did not change the primary chemical structure of the polymers. Hydrogen bonding interactions are suggested by slight variations in peak intensity and position. Ionic interactions between K^+^ ions and the sulphate groups of κ-carrageenan may be responsible for the slight variations in the spectra of KCl-crosslinked samples (sets C and D). The creation of hydrogel networks is facilitated by these interactions.

### 2.3. Porosity and Gel Fraction of Hydrogels

**Figure 3:**
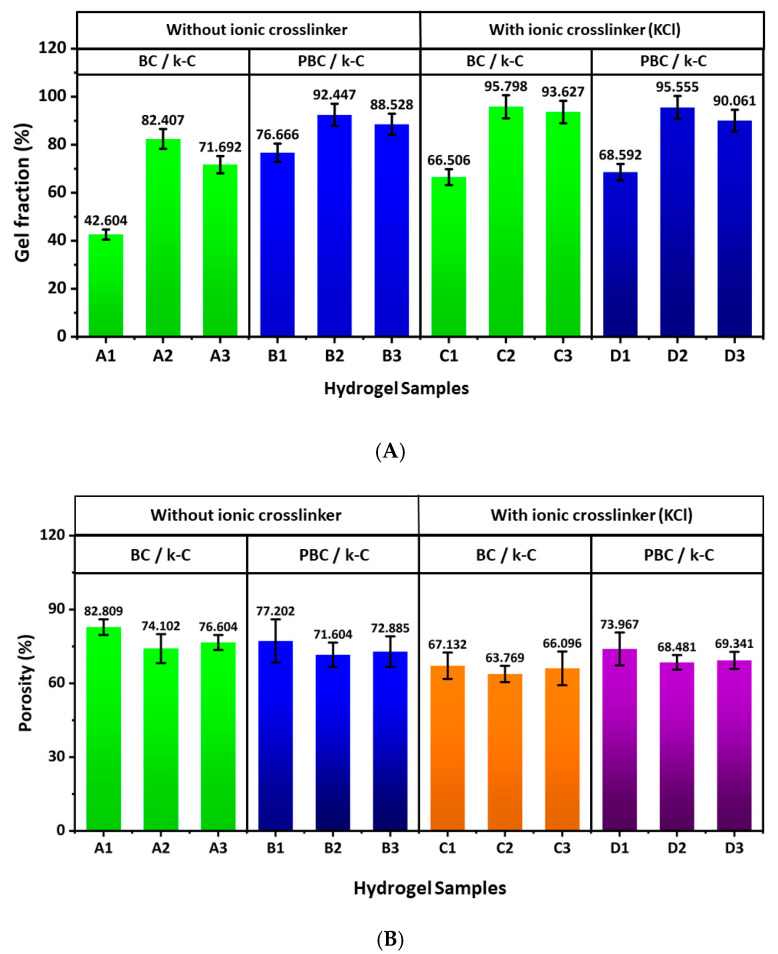
(**A**) represents the gel fraction, and (**B**) represents the porosity of BC/κ-carrageenan and PBC/κ-carrageenan hydrogels.

Figure 3A demonstrates the gel fraction of the hydrogel samples. For all 4 sets of hydrogels (A, B, C, and D), the gel fraction is higher in the second samples (A2, B2, C2, and D2). This can be justified by the proper crosslinking that occurs through the hydrogen bond formation between the hydroxyl group (–OH) of BC and the hydroxyl group (-OH) or sulphate (–SO_4_) group present in κ-carrageenan. In the case of C and D hydrogel samples, the gel fraction is slightly improved by the additional ionic crosslinking induced by K^+^ ions present in the κ-carrageenan solution, at room temperature [24,25,26].

The porosity of the hydrogel samples is shown in Figure 3B, which demonstrates that A2, B2, C2, and D2 have the lowest porosity across all A, B, C, and D sample sets, suggesting an inverse relationship between porosity and the gel fraction. This result can be attributed to a reduction in pore size resulting from greater crosslinking between the two polymers [27]. This data indicates that the gelation behaviour and pore size can be tuned by changing the polymer concentration during hydrogel formation for targeted applications.

### 2.4. Swelling Behaviour

**Figure 4:**
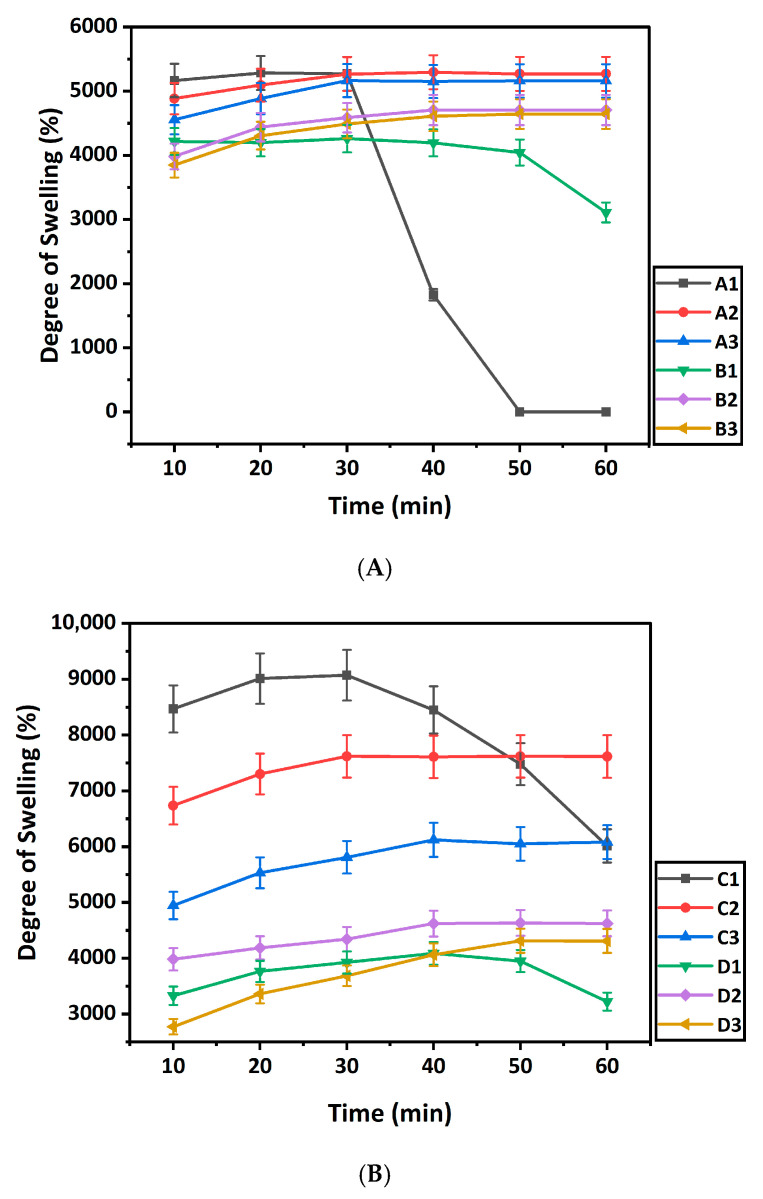

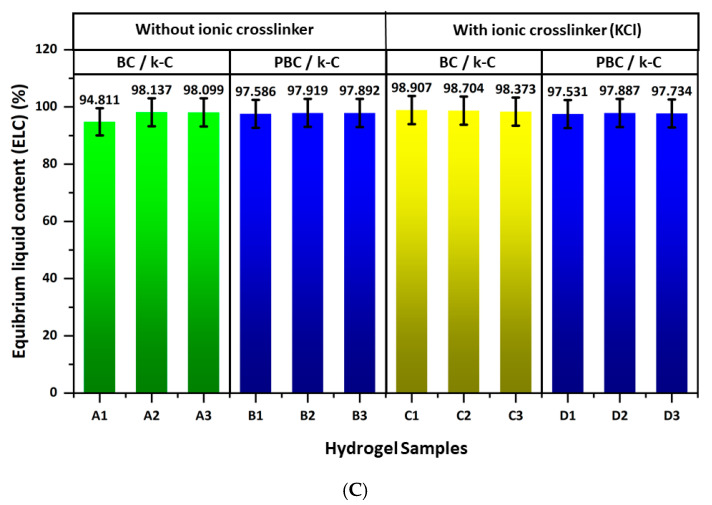
Degree of swelling of (**A**) BC/κ-carrageenan hydrogels, (**B**) PBC/κ-carrageenan, and (**C**) Equilibrium liquid content (ELC) of hydrogel samples.

Figure 4 demonstrates the swelling behaviour of the hydrogel samples developed with the different concentrations of BC/PBC and κ-carrageenan. Figure 4A shows the degree of swelling for samples without ionic crosslinking (A1–B3). The degree of swelling decreased with decreasing BC concentration in the samples A1, A2, and A3. The same result is observed for the samples B1, B2, and B3, where the degree of swelling decreased with the decrease of PBC concentration [28]. In the case of samples A1 and B1, the degree of swelling decreased due to poor crosslinking between the polymers and breakage and degeneration of the hydrogel structure [29]. The degree of swelling for sample A1 decreased sharply after 30 min, due to degradation of the hydrogel structure. This result is attributed to the lower concentration of κ-carrageenan in A1 (BC:κ-carrageenan = 2:1) compared to A2 (BC:κ-carrageenan = 1:1) and A3 (BC:κ-carrageenan = 1:2) hydrogel samples. Samples A2, A3, B2, and B3 reached equilibrium around 30–40 min of swelling, and still maintained their structure.

Figure 4B demonstrates the degree of swelling for samples with ionic crosslinking (C1–D3). A similar trend is observed for these hydrogel samples, with the degree of swelling decreasing as the BC and PBC concentrations decrease. Also, C1 and D1 show degeneration of the hydrogel structure resulting from the low crosslinking density. However, the rate of degeneration is slower in the case of C1 compared to A1 due to the presence of ionic crosslinking of the polymers in the C1 hydrogel. In addition, Figure 4C demonstrates that the equilibrium liquid content of all hydrogel samples exceeds 90%, confirming their high water-holding capacity and supporting their hydrophilic fluid absorption, facilitating potential tissue repair.

### 2.5. Antiseptic Solution Loading Capacity

**Figure 5:**
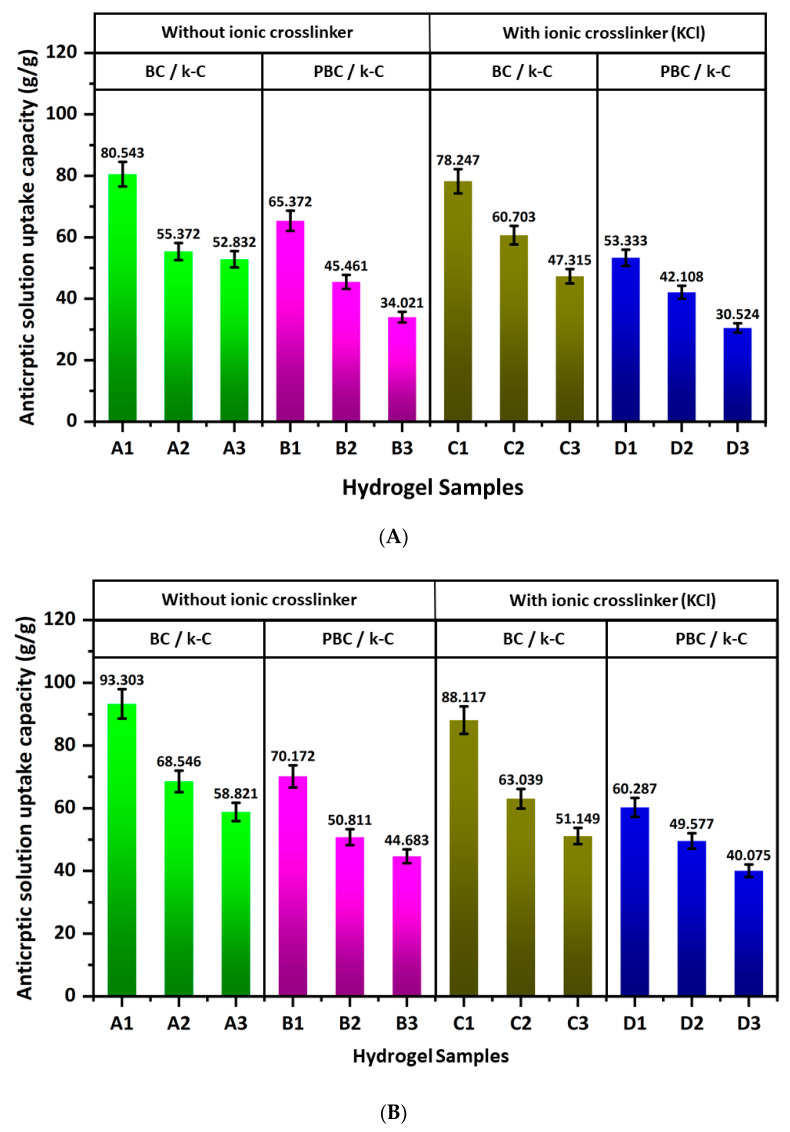

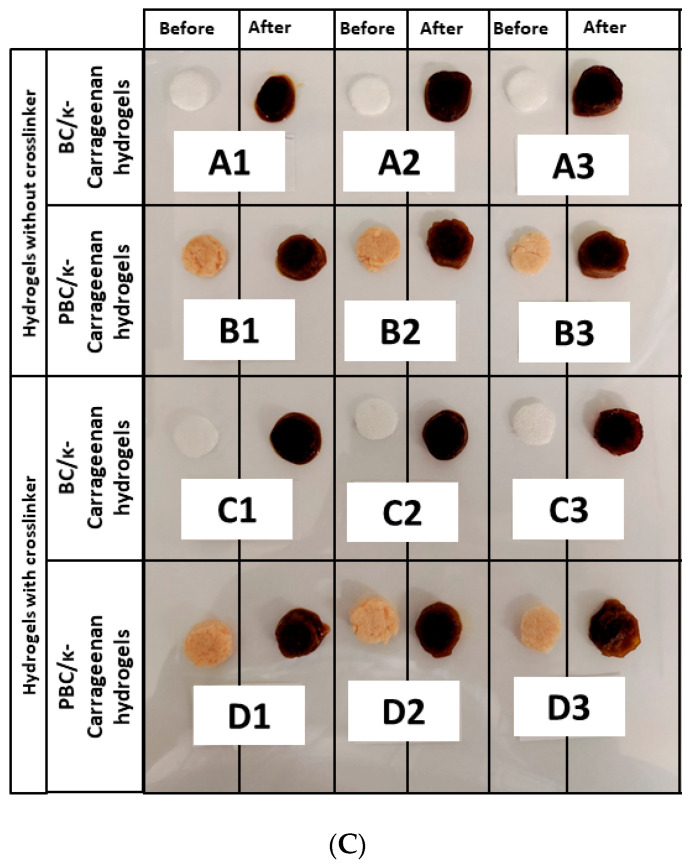
Antiseptic solution loading capacity of hydrogel samples for (**A**) 1:10 betadine solution and (**B**) 1:100 betadine solution, (**C**) change of colour of hydrogel samples after the absorption of antiseptic solution (betadine).

Figure 5 shows the antiseptic solution loading capacity of the hydrogel samples, where Figure 5A shows the loading capacity for a 1:10 betadine solution, and Figure 5B shows the loading capacity for a betadine solution of 1:100 concentration. The betadine loading capacity for both 1:10 and 1:100 betadine solutions is highest for the hydrogels with the highest BC and PBC concentrations and decreases gradually with increasing κ-carrageenan concentration. This result can be attributed to the hydrophilic nature of the BC structure and the increase in porosity related to BC concentration. Furthermore, the loading capacity is higher with a 1:100 betadine solution than with a 1:10 betadine solution, which can be attributed to the lower solution concentration, which supports faster absorption within the hydrogel network. Also, the results demonstrate lower loading capacity for C and D hydrogel samples compared to A and B, resulting from the ionic crosslinking in C and D hydrogel samples.

### 2.6. In Vitro Antiseptic Solution Release Kinetics

**Figure 6:**
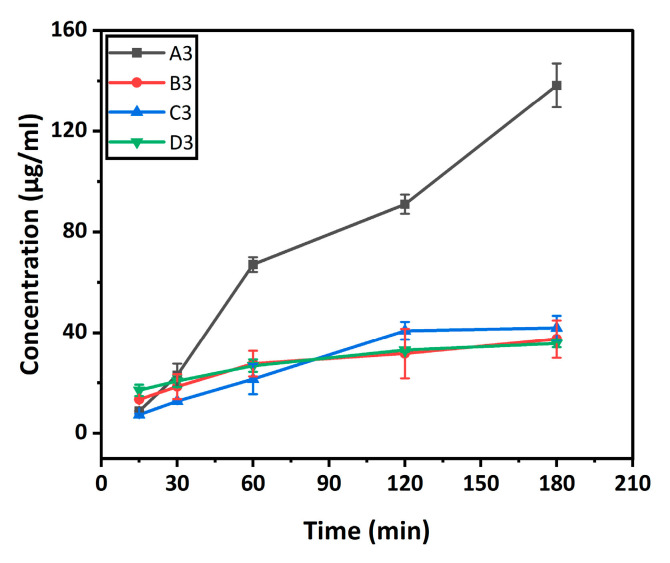
In vitro antiseptic solution release kinetics for BC/κ-carrageenan and PBC/κ-carrageenan Hydrogels.

The in vitro release kinetics of the betadine solution are shown in Figure 6. The release behaviour of povidone-iodine (Betadine) from hydrogel samples (A3, B3, C3, and D3) was evaluated in phosphate-buffered saline (PBS) over 180 min (Figure 6). The cumulative release profiles show a clear increase in Betadine concentration over time for all samples, indicating the successful loading of the antiseptic agent into the hydrogel matrices and its subsequent diffusion.

All formulations exhibited an initial rapid release within the first 30–60 min, followed by a more gradual, sustained release up to 180 min. This biphasic pattern is characteristic of hydrogel-based delivery systems, typically resulting from the immediate diffusion of surface-associated drug molecules (burst release), followed by controlled diffusion from the internal polymeric network.

A3 and C3 (BC/κ-carrageenan hydrogels) demonstrated better release compared to B3 and D3 (PBC/κ-carrageenan), which can be attributed to a larger amount of betadine loading during the swelling of hydrogel samples in betadine solution.

At every time interval, the non-crosslinked BC/K-carrageenan hydrogel (A3) exhibited the best release, reaching approximately 135 µg/mL after 180 min. This quick release is due to the comparatively loose network structure, which promotes quicker drug diffusion [30]. In contrast, the PBC/κ-carrageenan hydrogel without crosslinking (B3) demonstrated relatively reduced release (~40 µg/mL at 180 min), indicating that the addition of PBC creates a denser matrix that inhibits drug diffusion to some extent.

Introduction of KCl as an ionic crosslinker considerably changed the release behaviour. Compared to its non-crosslinked counterpart (A3), the crosslinked BC/K-carrageenan hydrogel (C3) exhibited more regulated and reduced release (~40 µg/mL at 180 min). Ionic interactions between potassium ions (K^+^) and the sulphate groups of K-carrageenan form a tighter polymeric network, resulting in smaller pores and fewer diffusion channels [31].

The crosslinked PBC/κ-carrageenan hydrogel (D3) exhibited the lowest release (~35 µg/mL at 180 min) of all the formulations, demonstrating the combined effects of ionic crosslinking and polymer modification on structural integrity. The denser network of D3 probably provides better resistance to drug diffusion and solvent penetration, resulting in a longer-lasting release profile.

Overall, the findings clearly show that ionic crosslinking and polymer composition are important factors in controlling drug release behaviour from hydrogel samples. The addition of PBC improves controlled release properties, while the inclusion of KCl considerably lowers the release rate.

### 2.7. Surface Morphology Analysis of BC/κ-Carrageenan and PBC/κ-Carrageenan Hydrogels

**Figure 7:**
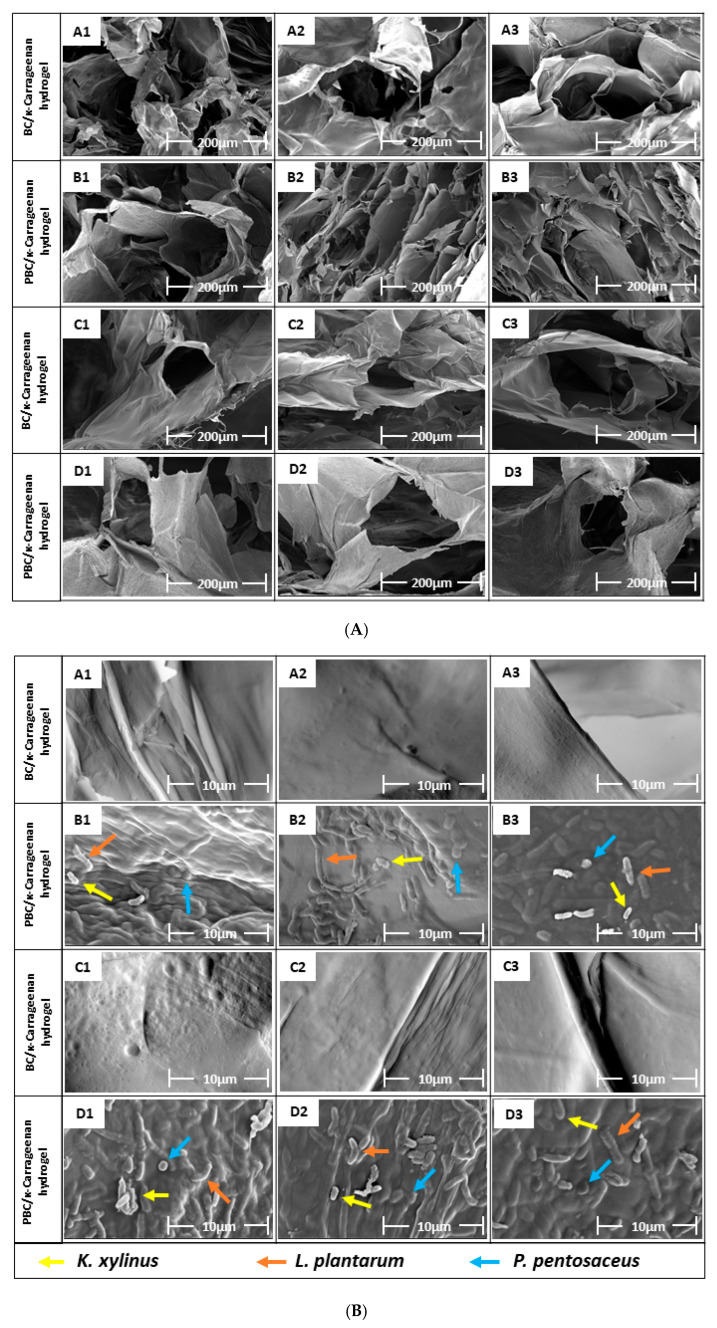
SEM micrograph of BC hydrogels, (**A**) porous microstructure of hydrogels, (**B**) presence of bacteria in the hydrogel structure.

The surface morphology and microstructure of the hydrogel samples were characterized by FE-SEM (Figure 7). The surface morphology is shown in Figure 7A, revealing an interconnected, porous 3D network that plays an important role in metabolite transport and nutrient exchange. The porous structure of the hydrogel network decreased with decreases in the BC and PBC concentrations [32]. A1, B1, C1, and D1 have the highest pore sizes due to higher concentrations of BC and PBC. A2, B2, C2, and D2 have the smallest pore sizes due to the highest crosslinking density among the three concentration variations [33], which contributes to the structural stability of the hydrogel. The increased structural stability of the hydrogel in comparison with the individual polymer and the inherent hydrophilic nature of the component polymers offer favourable conditions for cell migration and growth, which will make these hydrogels a potential candidate for biomedical applications.

Figure 7B demonstrates the presence of all three types of bacteria (*K. xylinus*, *L. plantarum*, and *P. pentosaceus*) as well as biofilm, produced by those bacteria, on the surface of B and D hydrogel samples due to the use of PBC. However, these bacteria were not found on the surfaces of the A and C sets of hydrogels due to the use of normal BC.

### 2.8. Mechanical Property

**Figure 8:**
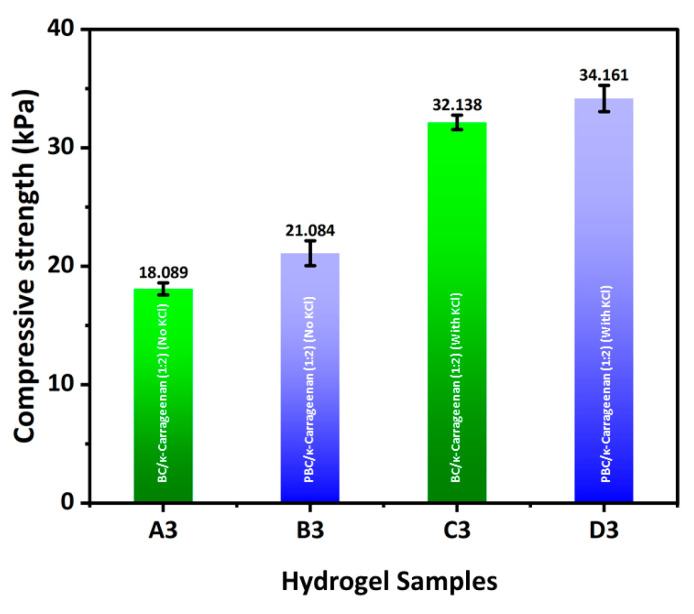
Compressive strength of BC/κ-carrageenan (A3, C3) and PBC/κ-carrageenan (B3, D3) hydrogel samples.

The compressive strength of BC/κ-carrageenan and PBC/κ-carrageenan hydrogel samples is demonstrated in Figure 8. A gradual increase in compressive strength was observed from A3 to D3. Hydrogel samples without crosslinking agent (KCl) (A3, B3) demonstrated lower compressive strength compared to the hydrogel samples with crosslinking agent (C3, D3). This result confirms that KCl crosslinking formed a better interconnected three-dimensional hydrogel network with improved mechanical integrity. However, incorporating PBC instead of BC into the hydrogel network improved its mechanical integrity. The compressive strength of all four hydrogel samples is observed within the range of human tissue (1–100 kPa) [34].

### 2.9. Antibacterial Assay

**Figure 9:**
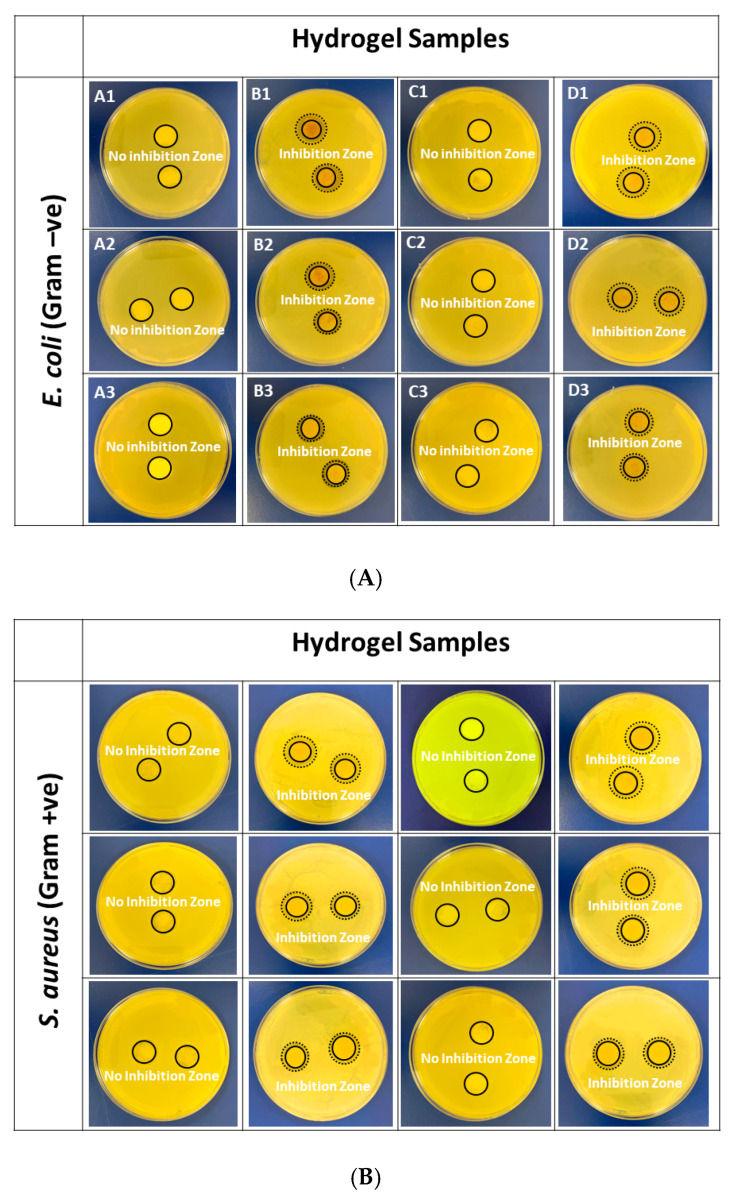
Antibacterial assay by agar disc diffusion method of BC/κ-carrageenan and PBC/κ-carrageenan hydrogels against common pathogenic bacteria (**A**) *E. coli* and (**B***) S. aureus.*

The antimicrobial assay of BC/κ-carrageenan and PBC/κ-carrageenan hydrogels was performed against the common pathogenic bacteria, *E. coli* and *S. aureus*, to examine their antibacterial and inhibitory activities against these bacteria, which are commonly present in infection environments [17,35]. Studies found that *L. plantarum* [36] and *P. pentosaceus* [37] exhibit both inhibitory and bactericidal properties against the Gram-positive *S. aureus* and the Gram-negative *E. coli.* Figure 9 demonstrates that both B and D hydrogel samples exhibited distinct inhibition zones against both pathogenic bacteria, *E. coli* and *S. aureus*. This result is attributed to the presence of PBC in B and D hydrogel samples, which includes the probiotic bacteria *L. plantarum* and *P. pentosaceus*. However, no inhibition zone can be found in the case of A and C samples due to the presence of BC instead of PBC. Moreover, the size of the inhibition zone decreases as the PBC concentration in the hydrogel samples decreases from B1 to B3 and D1 to D3. The size of the inhibition zone is shown in Table 1.

### 2.10. In Vitro Cell Viability

**Figure 10:**
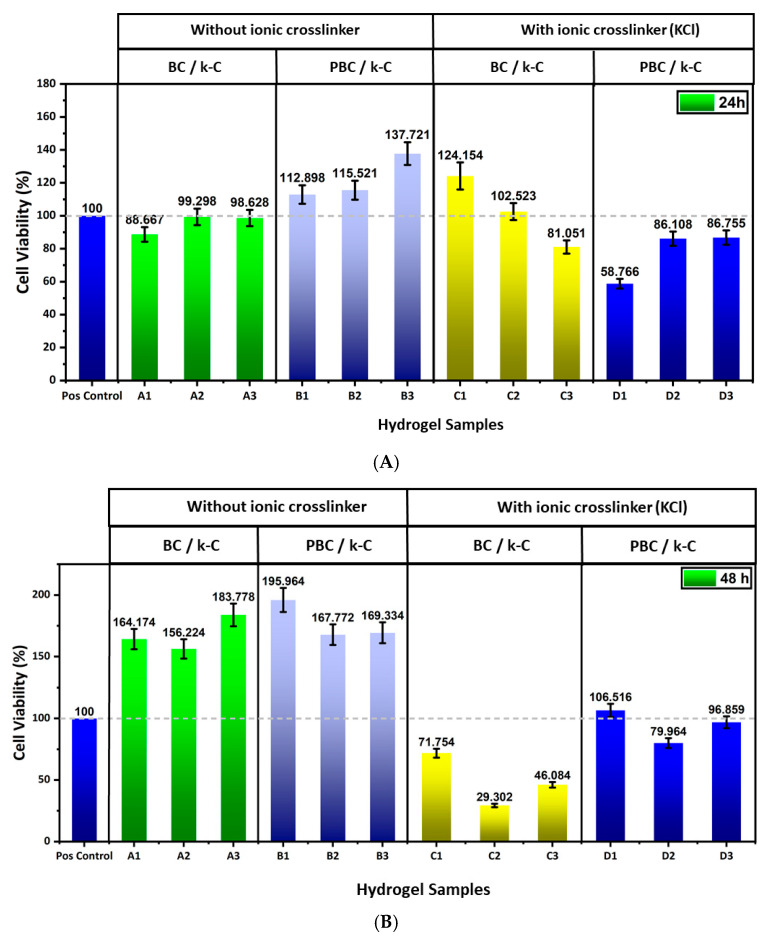
Cell viability assay of BC/κ-carrageenan and PBC/κ-carrageenan hydrogels by CCK-8 assay for (**A**) 24 h and (**B**) 48 h.

Figure 10 demonstrates the biocompatibility of BC/κ-carrageenan and PBC/κ-carrageenan hydrogels using the CCK-8 assay. After 24 h of exposure, set A hydrogels exhibited good biocompatibility. Similarly, set B hydrogels demonstrated higher cell viability values, indicating enhanced metabolic activity of the cells. Hydrogel samples of set C demonstrated variation in cell viability, where C and C2 showed cell viability of 124% and 102%, but C3 exhibited a reduced value in biocompatibility of 81%. However, set D hydrogels demonstrated lower biocompatibility values between 58–86%, where D1 exhibited the lowest value of 58%.

A different trend is observed in the case of 48 h of exposure. Set A hydrogels exhibited higher values in cell viability, suggesting strong support for fibroblast proliferation. Increased cell viability can be observed in the case of hydrogels of set B, indicating significant biocompatibility for the PBC-containing hydrogel systems. However, a significant decrease (72%, 29%, and 46% for C1, C2, and C3, respectively) can be observed in KCl crosslinked hydrogels of set C at a longer exposure time. Particularly, C2 showed significant cytotoxicity after 48 h of exposure. Set D hydrogels demonstrated moderate cytocompatibility (values ranging from approximately 80% to 106%), indicating improved cellular response in PBC-based systems compared to BC-based systems.

The result of the cell viability assay demonstrates that the polymer composition, as well as the ionic crosslinking, influenced the cell viability significantly. Hydrogel samples composed of BC and κ-carrageenan demonstrated good cell viability for BALB/3T3 cells at both time points, suggesting the combination of BC and κ-carrageenan offers a favourable environment for fibroblast growth [38,39]. The incorporation of PBC in set B hydrogels enhanced the cell viability (values exceeding more than 100%), which may be attributed to the hydrophilicity or nutrient diffusion from the biofilms produced by the probiotic bacteria in the PBC, which can enhance fibroblast proliferation. The introduction of KCl as an ionic crosslinker reduced the cytocompatibility. In the BC/κ-carrageenan hydrogel system, prolonged exposure resulted in a significant reduction in cell viability, indicating that higher crosslinking density or possible ion release affected the cellular metabolism over time. However, hydrogels of set D maintained moderate cell viability, suggesting that PBC stabilizes the hydrogel structure by regulating ion diffusion, thereby reducing potential cytotoxic effects.

These findings suggest that the presence of PBC in hydrogel systems enhances the cytocompatibility compared to BC, specifically at ionic conditions, indicating that the incorporation of PBC instead of BC in κ-carrageenan hydrogel systems makes them a promising candidate for biomedical applications.

## 3. Conclusions

In this work, a novel probiotic bacterial cellulose (PBC)/κ-carrageenan composite hydrogel was successfully developed and evaluated for its potential in biomedical applications. The incorporation of PBC into the hydrogel matrix introduced intrinsic antibacterial functionality, while κ-carrageenan enabled efficient gel formation and structural tunability. The visual observation of the developed PBC/κ-carrageenan appeared as light brown due to the presence of PBC. While BC/κ-carrageenan hydrogel appeared white due to the presence of pure BC. The physicochemical properties of the hydrogels, including swelling behaviour, porosity, gel fraction, and antiseptic solution (Betadine) loading capacity, could be modulated to a certain extent by adjusting the polymer composition and crosslinking conditions. The hydrogels exhibited high water uptake capacity and an interconnected porous structure, both desirable features for wound-dressing and tissue regeneration applications. The observed compressive strength of BC/κ-carrageenan (1:2) and PBC/κ-carrageenan (1:2) hydrogels was between 18–34 kPa (Figure 8), which is within the range of human tissue (1–100 kPa). In addition, these hydrogels demonstrated a relatively sustained release profile of betadine solution (Figure 6) over the investigated time period (15–180 min), indicating their potential for therapeutic delivery. Notably, PBC-containing hydrogels showed moderate antibacterial activity against both *Escherichia coli* and *Staphylococcus aureus*, whereas BC-based hydrogels exhibited no inhibitory effect. In vitro cytocompatibility studies further confirmed that PBC incorporation enhanced cell viability, although excessive ionic crosslinking reduced long-term cellular response.

Overall, among the prepared hydrogel samples, the PBC/κ-carrageenan hydrogel with a 1:2 PBC: κ-carrageenan ratio exhibits a synergistic combination of antibacterial activity, biocompatibility, and adjustable physicochemical properties within a moderate range. These findings highlight their potential as multifunctional biomaterials for wound healing and other tissue engineering applications. Future studies should focus on further optimization, in vivo evaluation, and the development of advanced therapeutic functionalities to further expand their clinical relevance.

## 4. Materials and Methods

### 4.1. Materials

The PBC was synthesized at the Centre of Polymer Systems (CPS) at Tomas Bata University in Zlin, Czech Republic. κ-carrageenan, Potassium Chloride (KCl), and absolute ethanol (EtOH) were purchased from Sigma-Aldrich, Czech Republic. Betadine antiseptic solution was purchased from a nearby pharmacy. All chemicals were used exactly as supplied, requiring no additional purification. Distilled water (dH_2_O) was used to prepare all solutions. Cell viability assay was performed using the CCK-8 kit (Sigma Aldrich, USA) with a complete cell medium, consisting of 1% penicillin/streptomycin (Sigma Aldrich, USA), 10% calf serum (Sigma Aldrich, USA), and 89% Dulbecco’s modified Eagle’s medium (DMEM) (Sigma Aldrich, USA).

### 4.2. Preparation of PBC/κ-Carrageenan Composite Hydrogel

PBC/κ-carrageenan Composite Hydrogel was prepared without using any crosslinker and by using potassium chloride (KCl) solution as an ionic crosslinker.

PBC was synthesized by co-culturing BC producing bacteria *Komagataeibacter xylinus* and probiotic bacteria *Lactobacillus plantarum* and *Pediococcus pentosaceus* in AJMRS media (apple juice:MRS media = 1:1). A homogeneous aqueous suspension of BC and PBC (1% *w*/*v*) was prepared by blending BC and PBC mats using the Kilig Vortex Portable Blender (Kilig, India) at 22,000 rpm for 2 min.

Two types of κ-carrageenan stock solution (2% *w*/*v*) were prepared. The first type of κ-carrageenan solution was prepared by dissolving κ-carrageenan in DI water at 80 °C, and the 2nd κ-carrageenan solution was prepared by dissolving κ-carrageenan in a 20 mM KCl solution at 80 °C.

PBC/κ-carrageenan hydrogels were prepared by homogeneous mixing of PBC and both types of κ-carrageenan solutions in 1:2, 1:1, and 2:1 ratios, separately. Also, normal BC/κ-carrageenan hydrogels were prepared by mixing normal BC and κ-carrageenan in a similar ratio with both types of κ-carrageenan solutions as control samples. After homogeneous mixing of both polymers, the hydrogel samples were cast in a 24-well cell culture plate and allowed to cool and form a gel at room temperature. All the hydrogels are shown in Table 2, along with the mixing ratios of the polymer suspensions and solutions.

All the hydrogel samples were freeze-dried for 24 h at −110 °C using a Labogene ScanVac CoolSafe Trigon plus freeze-dryer (Labogene, Denmark) and stored in a desiccator for further analysis [40].

### 4.3. Characterization

#### 4.3.1. Swelling Study

The swelling study was performed at 37 °C, using a phosphate-buffered saline (PBS) (pH 7.4). The hydrogel samples were soaked in PBs Solution and measured at specific intervals (10, 20, 30, 40, 50, and 60 min) until swelling equilibrium was reached [17]. The degree of swelling of the hydrogel samples was measured by the Equation (1):

Degree of swelling (%) = [(W_s_ − W_d_)/W_d_] × 100 (1)

where W_s_ and W_d_ are denoted as the weights of the hydrogel samples in swollen and dried conditions, respectively.

The equilibrium liquid content (ELC) of the hydrogel samples, where PBS is used as the liquid was calculated [17] by the Equation (2):

Equilibrium Liquid Content (ELC) (%) = [(M_s_ − M_0_)/M_s_] × 100 (2)

where M_s_ and M_0_ are the swollen weight of the hydrogel samples at equilibrium and the initial weight of the dry hydrogel samples, respectively.

Following the swelling experiment, the hydrogel samples were reweighed after drying in a vacuum oven until constant weight [41]. The following equation was used to get the gel fraction (Equation (3)):

Gel fraction (%) = (M_d_/M_0_) × 100 (3)

where, M_d_ and M_0_ are the weights of the hydrogels after drying (2nd dry weight) and before swelling (1st dry weight), respectively.

All the results are collected in triplicate, and the results are presented by average values.

#### 4.3.2. Porosity and Antiseptic Solution Uptake Capacity

The porosity percentages of the PBC/κ-carrageenan hydrogel samples were determined using the liquid-displacement method. All of the hydrogel samples [sample size (V_1_): 400–800 mm^3^; diameter: 14 mm, height: 3–5 mm] were soaked with absolute ethanol for 4 h at 37 °C, until reaching absorption saturation. The porosity (%) of the hydrogel samples was calculated [17] by the following Equation (4):

Porosity (P) = (W_2_ − W_1_)/ρV_1_ (4)

where W_1_ and W_2_ are the weights of the hydrogel samples before and after immersion in absolute ethanol, respectively. V_1_ is the volume of the hydrogels before their immersion in ethanol, and ρ (0.00078691 g/mm^3^ at 37 °C) is the constant of the density of ethanol.

The antiseptic solution uptake capacity of the hydrogel samples was evaluated by determining the absorption of antiseptic solution (betadine) by the freeze-dried hydrogel samples. Antiseptic liquid (Betadine) solution was prepared in a 1:10 and 1:100 ratio of Betadine and DI water. The freeze-dried hydrogel samples were soaked in both of the betadine solutions until the equilibrium is reached. The antiseptic solution loading capacity was then calculated by using the following Equation (5).

Antiseptic solution loading capacity (g/g) = (W_s_ − W_d_)/W_d_ (5)

where W_d_ and W_s_ are the weights of the dried and swollen hydrogels after soaking the betadine solution, respectively.

All the results are collected in triplicate, and the results are presented by average values.

#### 4.3.3. In Vitro Antiseptic Solution Release Kinetics

Antiseptic liquid (Betadine) release kinetics were studied using a 10% betadine solution. All the hydrogel samples were allowed to swell in 10% betadine (povidone-iodine) solution until equilibrium was reached. Thereafter, the betadine-swollen hydrogel samples were placed in 10 mL of PBS and allowed betadine to be released from the hydrogels. A total of 2 mL of PBS was collected at specific intervals of time (15, 30, 60, 120, and 180 min) and kept for UV–Vis spectrophotometry. The 2 mL of PBS was added to each beaker immediately after collecting the sample to maintain the sink condition. Samples from only A3, B3, C3, and D3 were measured at 288 nm [42,43] using a SPECORD 250 Plus (Analytic Jena AG, Germany) UV–Vis spectrophotometer, as the rest of the betadine-soaked hydrogel samples started degrading after placing them in PBS solution. All the results are collected in triplicate, and the results are presented by average values. The concentration of betadine in the PBS solution at each time point was measured by using a calibration curve equation derived from the linear regression analysis.

The standard curve for betadine was prepared using a SPECORD 250 Plus (Analytic Jena AG, Germany) UV–Vis spectrophotometer. A working stock solution was prepared by diluting Betadine in a 1:100 ratio with PBS. Serial dilutions were prepared using 0.5, 1.0, 1.5, 2.0, and 2.5 mL of the working stock solution, then brought to 10 mL with PBS. The absorbance of each betadine solution was measured at 288 nm [43], where PBS was used as a blank. A standard curve was prepared by plotting absorbance vs. concentration, and linear regression analysis was performed.

#### 4.3.4. Structural Analysis

Fourier transform infrared (FTIR) spectra of the hydrogel samples were analysed to observe the functional groups present in the hydrogel samples and were documented by using a Nicolet iS5 (Thermo Scientific, USA) by scanning at a resolution of 4.0 cm^−1^ between the range of 4000 to 400 cm^−1^.

The BC/κ-carrageenan and PBC/κ-carrageenan hydrogel samples were analysed for their morphological microstructure by using a SEM (SEM, FEI, Czech Republic), where the accelerating voltage was 5 kV. Prior to the analysis, the hydrogel samples were freeze-dried, followed by Au/Pd coating using an auto fine coater by JEOL JFC 1300 (JEOL Ltd. Tokyo, Japan) to improve surface conductivity.

#### 4.3.5. Mechanical Test

The compressive strength of BC/κ-carrageenan and PBC/κ-carrageenan hydrogels was measured using an Instron testing machine (Model 8871, UK) at room temperature (25 °C) by using compression mode [44]. The samples were positioned on top of a cylindrical metal disc to perform the compression. A load cell with a 10 N capacity and a speed of 5 mm/min was used for the compression study. Only the A3, B3, C3, and D3 samples were measured and presented, as the rest of the samples were too soft for reliable measurement.

Compressive strength of the hydrogel samples was determined from the load displacement curve at the point of break. The results were displayed as an average value after the samples were measured in triplicate.

#### 4.3.6. Antibacterial Assay

The antibacterial activity of all BC/κ-carrageenan and PBC/κ-carrageenan hydrogels was investigated against *E. coli* and *S. aureus* using the agar disc diffusion method [17,35]. A 0.1 mL aliquot of each bacterial stock (approximate concentration 14 × 10^8^ CFU/mL) was uniformly spread on TSA plates. The freeze-dried hydrogel samples were cut into a disc shape of a 14 mm diameter and were placed on the agar plates containing the bacterial suspension. After incubation for 24 h at 37 °C, antimicrobial activity was evaluated by observing the zone of inhibition formed around the hydrogel samples.

The results (diameter of zone of inhibition) are measured in triplicate, and the results are presented as average values.

#### 4.3.7. In Vitro Cell Viability

Murine fibroblast cells (BALB/3T3), obtained from the cell culture facilities of the Baltic Biomaterials Centre of Excellence (Riga, Latvia), were used to evaluate the cytocompatibility of the hydrogels via an indirect extraction method. This experiment was performed at the Baltic Biomaterials Centre of Excellence (Riga, Latvia). The hydrogels were first sterilized under UV light for 30 min on both sides of the hydrogel samples. Hydrogel extracts were prepared by incubating sterilized hydrogels (50 mg/mL) in complete culture medium composed of Dulbecco’s Modified Eagle Medium (DMEM) supplemented with 10% calf serum and 1% penicillin/streptomycin. The hydrogels were incubated in the medium for 24 and 48 h at 37 °C, after which the extracts were collected and stored at −20 °C until further use.

BALB/3T3 cells were cultured in standard conditions (37 °C, 5% CO_2_, and humidified atmosphere). Upon reaching approximately 80% confluence, the cells were washed using PBS and detached by using trypsin. The cells were then counted and seeded into 96-well plates at a density of 5 × 10^3^ cells per well and allowed to adhere overnight.

Following the cell attachment, the culture medium was removed and replaced with the prepared hydrogel extracts and the cells were incubated for 24 h. Afterwards, 10 μL of CCK-8 reagent was added to each well containing 100 μL of culture medium, and the plates were incubated for 2 h at 37 °C. The absorbance was measured at 450 nm using a microplate reader. Cell viability was expressed as a percentage relative to the control group. All the results are collected in triplicate, and the results are presented by average values.

## Figures and Tables

**Table 1:** Zone of inhibition for BC/κ-carrageenan and PBC/κ-carrageenan hydrogels against *E. coli* and *S. aureus*.

		Pathogenic Bacteria	
		*E. coli* (Gram −ve)	*S. aureus* (Gram +ve)
**Hydrogel Samples**	A1	0	0
	A2	0	0
	A3	0	0
	B1	8.33 ± 0.47	8.67 ± 0.81
	B2	4.67 ± 0.66	6.33 ± 0.47
	B3	2.67 ± 0.81	3.33 ± 0.66
	C1	0	0
	C2	0	0
	C3	0	0
	D1	8.67 ± 0.57	7.67 ± 0.66
	D2	5.67 ± 0.81	5.33 ± 0.57
	D3	3.67 ± 0.47	2.67 ± 0.81

**Table 2:** BC/κ-carrageenan and PBC/κ-carrageenan Composite Hydrogel composition with polymer ratio.

Sample Index	Biopolymers				
		BC	PBC	κ-Carrageenan	
**A1**	**BC/κ-carrageenan Hydrogels**	2	-	1	**Without Crosslinker (KCl)**
**A2**		1	-	1	
**A3**		1	-	2	
**B1**	**PBC/κ-carrageenan Hydrogels**	-	2	1	
**B2**		-	1	1	
**B3**		-	1	2	
**C1**	**BC/κ-carrageenan Hydrogels**	2	-	1	**With Crosslinker (KCl)**
**C2**		1	-	1	
**C3**		1	-	2	
**D1**	**PBC/κ-carrageenan Hydrogels**	-	2	1	
**D2**		-	1	1	
**D3**		-	1	2	

## Data Availability

The original contributions presented in this study are included in the article. Further inquiries can be directed to the corresponding author.

## Abbreviations

The following abbreviations are used in this manuscript:

ECM	Extracellular Matrix
BC	Bacterial Cellulose
PBC	Probiotic Bacterial cellulose
FTIR	Fourier Transform Infrared
SEM	Scanning Electron Microscopy
ELC	Equilibrium Liquid Content
DMEM	Dulbecco’s Modified Eagle Medium
